# Examining child stress response, parenting and relationship-related changes associated with a dyadic mindfulness and self-compassion program

**DOI:** 10.3389/fpsyg.2026.1933287

**Published:** 2026-10-15

**Authors:** Christine R. Lathren, Jessica L. Borelli, Rosemary Ellsworth, Elondra Harr, Samantha DiMaio, Jamie Lynn Tatera, Veronica Diaz, Karen Bluth, Ryan J. Herringa, Sara A. Heyn

**Affiliations:** 1 Department of Physical Medicine and Rehabilitation, University of North Carolina at Chapel Hill, Chapel Hill, NC, United States; 2 Department of Psychology and Social Behavior, University of California, Irvine, Irvine, CA, United States; 3 Department of Public Health Education, University of North Carolina at Greensboro, Greensboro, NC, United States; 4 Department of Psychiatry, University of Wisconsin School of Medicine & Public Health, Madison, WI, United States; 5 Wholly Mindful, Milwaukee, WI, United States; 6 Department of Psychiatry, University of North Carolina at Chapel Hill, Chapel Hill, NC, United States; 7 Medical College of Wisconsin, Milwaukee, WI, United States; 8 Department of Psychiatry and Behavioral Medicine, Medical College of Wisconsin, Milwaukee, WI, United States

**Keywords:** dyadic, mindfulness, parent–child, school-aged, self-compassion, stress

## Abstract

**Introduction:**

Interventions that dyadically equip families with self-compassion skills represent a promising approach for addressing mental health challenges, particularly during the key transitional period of middle childhood. This study examines changes and candidate processes associated with participation in the Mindfulness and Self-Compassion for Children and Caregivers (MSC-CC) course.

**Methods:**

Twenty-three caregiver-child dyads (Child, *M*_age_=8.78; Caregiver, *M*_age_=41.2) participated in MSC-CC, delivered remotely over six weekly group sessions. Pre- and post-intervention assessments included self-report measures of parenting and relational closeness outcomes during a dyadic stressor task (behavior, reflective functioning[RF], and physiology). Analyses investigated pre-post change in primary outcomes and linear mixed-effects models estimated time-related changes and baseline moderators of physiological change. Significance tests identify pre-post increases in parenting and emotion-supportive beliefs.

**Results and Discussion:**

Physiologically, we detected significant decreases in child physiological arousal. Exploratory moderation analyses suggest, among others, low levels of mindfulness and high RF at baseline predict the largest reductions in child stress physiology. While uncontrolled, the MSC-CC participation was associated with increases in mindful parenting and the caregiver-child relationship and lower child physiological arousal during a dyadic challenge, most prominently in families with lower emotion-supportive beliefs at baseline. Altogether, this highlights candidate processes and moderators of efficacy to be confirmed in a fully powered randomized controlled trial.

**Trial registration:**

ClinicalTrials.gov, *Self-Compassion for Children and Caregivers (SCCC)*, NCT05818397, registration and study start on 2023-10-16.

## Introduction

Childhood is a critical time for both physical and mental growth, particularly their ability to regulate their own emotions and stress response systems mature. Persistent dysregulation stress in childhood, often marked by heightened physiological responses to chronic stress, can increase vulnerability to psychopathology (Boyce et al., 2001; Calkins et al., 2007). Fortunately, a strong parent–child relationship can act as a protective buffer (Feldman, 2017; Miller et al., 2023), particularly during middle childhood (i.e., the preadolescent stage, ages 8–11) (Suveg et al., 2019). This key transitional period toward adolescence is characterized by increased emotional awareness and malleable emotion regulation skills (Liu et al., 2022; Antony et al., 2025) and may be particularly responsive to parent–child relational interventions that provide protection through the transition to high school, late adolescence, and even adulthood. Recent reports emphasize the exigency of novel, early intervention approaches during this period, reporting almost 40% of high school students experienced persistent feelings of sadness or hopelessness in the past year and 20.4% seriously considering suicide (Centers for Disease Control and Prevention, 2020).

Self-compassion (SC), defined as the practice of being supportive to oneself when experiencing difficult emotions, has been identified as a teachable emotion-regulation resource for children and caregivers (Neff and McGehee, 2010). The six primary components of self-compassion, representing positive and negative poles of three main dimensions, include: (1–2) mindfulness, or maintaining a balanced perspective amidst distress, and corresponding low levels of emotional avoidance or overwhelm; (3–4) self-kindness, or maintaining a supportive and understanding attitude towards oneself, and corresponding low levels of harsh self-judgment; and (5–6) common humanity, or recognizing that difficult emotions are a normal and universal part of the human experience, and corresponding low levels of isolation (Neff and McGehee, 2010). By cultivating these domains, both children and caregivers can fundamentally shift their relationship with distress (Marsh et al., 2017; García-Campayo et al., 2024), which has significant implications for their overall psychological well-being (Liu, 2015).

Self-compassion skills in both children and caregivers have been robustly linked to positive mental health outcomes and parenting. Higher levels of self-compassion are associated with lower psychological distress, anxiety, and depression in children (Marsh et al., 2017) and lower parenting stress, greater emotional regulation, and higher levels of mindful parenting in caregivers (Nguyen et al., 2020; Psychogiou et al., 2016). When faced with a child’s difficult emotions, mindful parenting fosters emotionally aware attitudes and behaviors that are nonjudgmental and supportive. Given the reciprocal nature of emotion regulation in families and the positive impacts on all members within a family structure, teaching self-compassion within a child and caregiver dyadic unit may improve outcomes with lower time-intensiveness for both individuals and therapists. Research in other clinical domains has already indicated that simultaneous parent–child interventions often yield more synergistic and sustainable outcomes than individual treatments alone (Everett et al., 2021). While largely untested, a dyadic delivery of SC programming to children and primary caregivers may facilitate more robust individual and relational changes using a comparatively quicker and more convenient approach.

Mindfulness and Self-Compassion for Children and Caregivers (MSC-CC) is a pilot program developed to teach caregiver-child dyads mindfulness and self-compassion skills using developmentally appropriate and engaging material for school-aged kids. Preliminary analyses within the same cohort have been reported separately, indicating that MSC-CC participation was feasible and acceptable and was associated with increases in child self-compassion skills and improvements in caregiver-reported child mental health outcomes (Lathren et al., 2026). However, the specific mechanisms that drive these improvements remain unknown. If we can identify candidate pathways through which intervention occurs, we are better equipped to optimize program delivery and determine which families will benefit most from specific components, leading to individualized and targeted treatment delivery. The current study examines, within the same cohort, a distinct set of outcomes not reported elsewhere across three general categories of factors that may play a role in child mental health improvements: (1) the child’s physiologic response to a dyadic stressor, (2) the caregiver’s behavioral and reflective response to stress, and (3) broader parent–child relational factors.

First, increased child SC skills may improve child mental health by modulating the intensity and duration of the physiological stress response. The stress response is defined as the activation of the autonomic nervous system (ANS) in reaction to perceived challenges, characterized by a dynamic balance between sympathetic arousal and parasympathetic recovery (Oubaid, 2023). More efficient physiological regulation, often seen as a dampened or more flexible response to stressors, is a key indicator of psychological resilience (De Los Reyes et al., 2011). The ability to use the stress response as a robust and unbiased proxy of mental health is a particularly powerful tool in pediatric populations with higher rates of informant discrepancies in internalizing symptoms (i.e., child versus caregiver reports) (De Los Reyes et al., 2011). To do so, we can quantify ANS function during challenging tasks using non-invasive markers like heart rate variability (HRV), or the variation in the time interval between heart beats (Appelhans and Luecken, 2006), and skin conductance, which tracks sweat gland activity as a proxy of emotional arousal (Boucsein, 2012).

Next, dyadic self-compassion (SC) programming may promote adaptive caregiving by mitigating the negative impact of stress on warmth, attunement, and flexibility (Barreto et al., 2024; Ward and Lee, 2020). While parenting-related stress and anxiety often lead to overreactive or controlling behaviors (Borelli et al., 2014a; Van Dijk et al., 2023; Sadoughi, 2024), SC may help caregivers regulate their emotions and respond with support rather than reactivity (Paucsik et al., 2023). Furthermore, by reducing defensive reactions, SC enhances the ability to understand the mental states of oneself and others (i.e., reflective functioning, RF) (Borelli et al., 2021). This nonjudgmental awareness encourages curiosity about complex emotions, ultimately fostering greater parenting sensitivity, healthier socioemotional development, and secure attachment for the child (Stern et al., 2015; Borelli et al., 2016).

Finally, while existing evidence suggests caregiver self-compassion is linked to positive parenting factors such as self-reported mindful parenting practices (e.g., noticing emotional states in oneself and one’s child) (Fernandes et al., 2023) and indicators of relational closeness (van Heerden et al., 2025), we aim to extend these findings by exploring if learning self-compassion is associated with increases in a caregiver’s adaptive beliefs about their child’s emotions. Studies suggest that caregivers who believe emotions are valuable, manageable, and not used to manipulate tend to coach emotions more effectively and model emotion regulation for their children (Dunsmore et al., 2013; Cenușă et al., 2023). When caregivers practice self-compassion, they shift away from judgmental, overidentified, or avoidant responses to their own and their children’s emotional states. It is possible that these adaptive beliefs in turn shape how caregivers interpret and respond to children’s emotional expressions, supporting children’s positive mental health.

Here, we extend the previous work with the MSC-CC intervention (Lathren et al., 2026) by examining changes in self-reported child and caregiver relational measures (parenting stress, relational closeness), reflective functioning and caregivers’ observed behavior and child stress physiology during a dyadic stress task measured before and after intervention participation. As an exploratory aim, we also examine if baseline values of any of these variables moderate time-related change in the child’s stress physiology. *A priori* hypotheses predicted that MSC-CC participation would be associated with increased dyadic closeness, mindfulness in parenting, and decreased parenting stress, alongside increases in caregiver reflective functioning. We further predicted decreases in the child’s physiological stress response (i.e., lower SCL and higher HRV) and lower caregiver overcontrol and higher caregiver support during the dyadic stress task. Our findings will guide the development of protocols for a larger, randomized control trial of MSC-CC participation investigating effectiveness and individualized prediction of treatment response.

## Methods

### Cohort recruitment and study procedures

Twenty-three school-aged children (ages 8–11, inclusive) and accompanying caregivers completed a pre-post pilot intervention study of the Mindfulness and Self-Compassion for Children and Caregivers (MSC-CC) course. Participants were recruited across two cohorts (Cohort 1, *n* = 11; Cohort 2, *n* = 12) and cohort size was capped at 12 dyads to facilitate comfort, connection, and participation in an online group intervention. Dyads were primarily recruited in Madison, WI from local elementary schools through direct staff referrals, recruitment events, and physical and electronic flyers. Participants were eligible if the child was aged 8–11, they had access to internet-enabled devices (hot spots were provided, when needed), and they were both proficient in reading, writing, and communicating in English. Dyads were excluded from participating if the child had active psychosis, a medical condition that might impair participation, or was currently taking a psychotropic medication known to directly affect sympathetic nervous system function. An elevated symptom level (child or caregiver report on the PROMIS Anxiety or Depression measures yielding a *T*-score greater than 60 at the time of enrollment) was an additional eligibility requirement for Cohort 2 (*n* = 12) only, included to broaden the distribution of child psychopathology symptoms in the study sample (Irwin et al., 2010). Across the full sample, 20 of 23 dyads met this elevated-symptom threshold via at least one measure (caregiver-reported depression, *N* = 16; caregiver-reported anxiety, *N* = 15; youth self-report depression, *N* = 7; youth self-report anxiety, *N* = 7 dyads meeting criteria via more than one measure, *N* = 14). Study procedures were completed in accordance with the study protocol approved by the Health Sciences Institutional Review Board at the University of Wisconsin-Madison (2023–0325). All participating caregivers and children provided informed consent and assent, respectively, prior to beginning study activities. This study is registered with clinicaltrials.gov (NCT05818397; registration and study start on 2023-10-16), which reflects a target enrollment of 25 dyads, where actual enrollment was 23 dyads, all of whom were included in these analyses and the companion publication. Any data related to the Spanish-speaking MSC-CC intervention arm of this study are not included here.

Study procedures and outcomes of interest are summarized in Figure 1. In summary, interested families completed an online web screen to determine study eligibility. After consent and assent procedures, children and caregivers independently completed a series of self-report questionnaires assessing parenting beliefs and the parent–child relationship followed by a dyadic repeated stressor task (Performance Challenge Task, details below). Enrolled families were invited to an optional online orientation, which allowed families to meet each other and the facilitators without providing any critical intervention programming, followed by the six-week Mindfulness and Self-Compassion for Children and Caregivers (MSC-CC) intervention. Here, for an hour weekly, the group cohort of parent–child dyads met remotely with 1–2 course facilitators. One to two weeks after course completion, the dyad was brought back to the lab to complete an identical set of questionnaires and the stressor task. Dyads received financial compensation for their time and effort at each laboratory visit.

**Figure 1:**
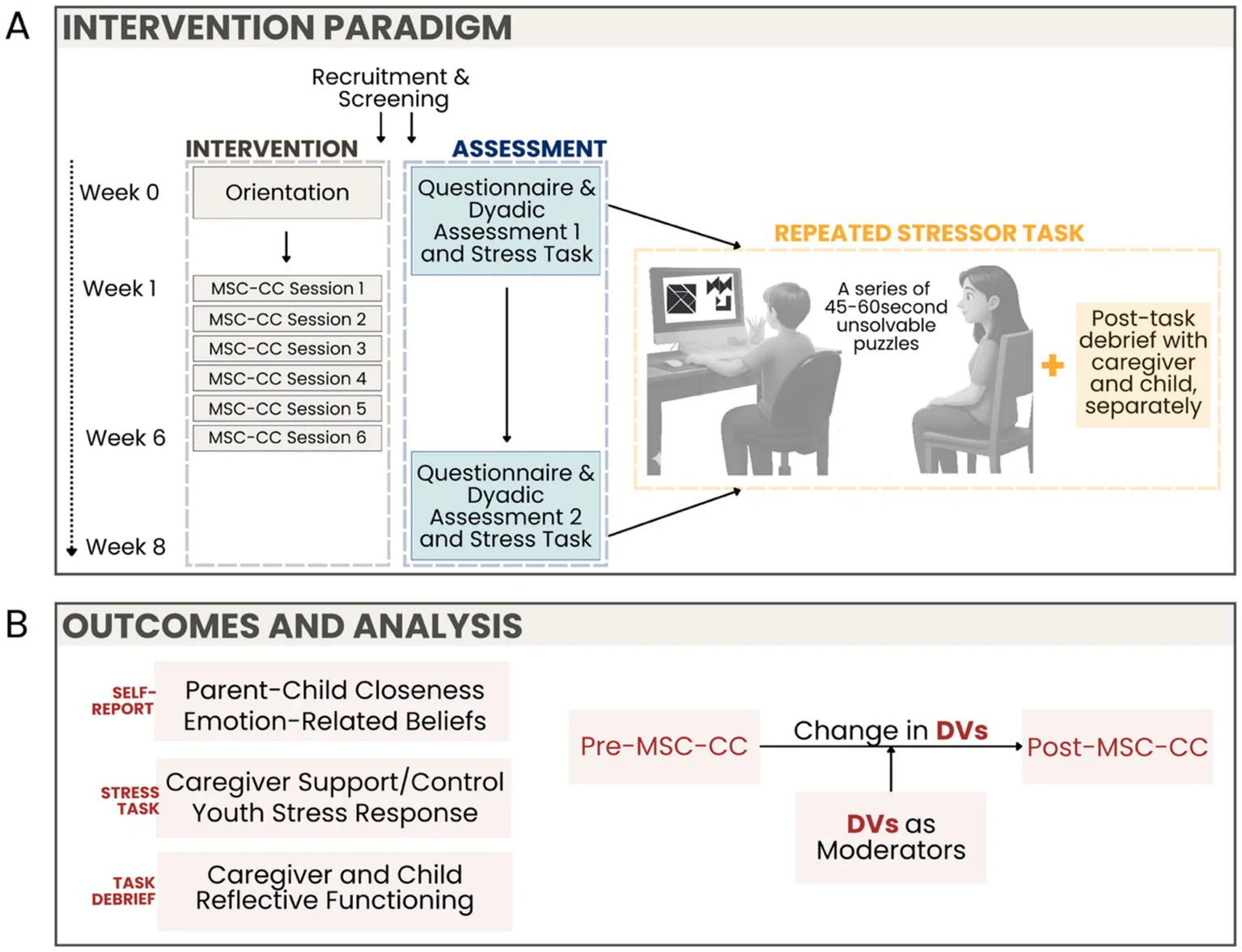
Planned outcomes and analyses of change associated with the Mindfulness and Self-Compassion for Children and Caregivers (MSC-CC) intervention. (A) Graphic showing study participant timeline, repeated stressor task details, and (B) Outcomes of interest (i.e., dependent variables, DVs), and analytical plan. DVs were grouped by self-reported questionnaires, measures derived from the stressor task, and measures derived from the post-task debrief. Planned analyses included pre-post change in DVs and DVs as moderators of change.

### Mindfulness and self-compassion for children and caregivers (MSC-CC)

Mindfulness and Self-Compassion for Children and Caregivers (MSC-CC) is a 6-week live online group course, consisting of weekly one-hour sessions. As an adaptation of the adult Mindful Self-Compassion (MSC) program, it was modified to engage elementary school-aged children and their caregivers through child-friendly methods, including movement, games, props, comics, and dyadic discussion topics. Further details regarding intervention development, facilitator training, session attendance, retention, and fidelity monitoring within this cohort are available elsewhere (Lathren et al., 2026) and summarized here. The course retains MSC’s core conceptual structure of introducing mindfulness and self-compassion skills prior to applying them to difficult emotions, while adapting component into concrete, child-friendly language and materials, including movement, games, props, and comics. Specifically, MSC-CC is structured around five resilience habit animals that represent core skills: Spots (noticing feelings, thoughts, body sensations, and five senses), Buddy (common humanity, or remembering difficult emotions happen to all humans, and we are not alone when we have them), Super Snuggles (self-kindness, or kind, encouraging self-talk), Doodles (meeting needs, or taking actions to support oneself), and Sunny (savoring good things). Each session was led by 1–2 certified MSC facilitators, who encouraged caregivers and children to mute themselves and practice compassionate/self-compassionate responding with each other during moments in the course. To reinforce learning, caregivers received weekly reminders of content and home practice options, including activity sheets from Volumes 1 and 2 of the *Mindfulness and Self-Compassion Workbook for Kids*. A full breakdown of weekly course content can be found in Supplemental Table 1.

### Dyadic repeated stressor task

For this study, we used a dyadic repeated stressor task, called the Performance Challenge Task (PCT). This is a standardized in-lab stress task that assesses physiologic, behavioral and psychological responses to a challenging and stressful experience for child-caregiver dyads (Borelli et al., 2014b; Borelli et al., 2018; Sichko et al., 2016). Details of this task are extensively reported elsewhere (Borelli et al., 2018) and briefly summarized here. The child participant sat at a computer to complete a series of impossible puzzles while the caregiver sat in a chair 6 feet behind the computer, a position that facilitated watching and supporting verbally but not close enough to move the mouse and physically help with the puzzle. The child and caregiver were given instructions, with no explicit directives given to the caregiver regarding their role in the game, followed by a practice round. Next, the child completed a series of six 45-s to 1-min (second and first cohort, respectively) trials of a tangram-style puzzle. During each trial, the child would rotate and place individual pieces to match the target configuration. Dyads were not informed that the puzzles were impossible to solve and the research team member led them to believe that each puzzle had a solution and that other children generally complete them in the allotted time. When the timer ran out, a screen appeared showing a bar graph indicating the number solved versus unsolved (i.e., showing none solved). To minimize the possibility of pattern recognition, the pre- and post-intervention stressor tasks utilized novel puzzle sets. Finally, after the first cohort, each puzzle trial was decreased in time (from 1-min to 45-s) and two solvable puzzles were randomly added to the trial list, as some children (noted in approximately 29% of videos) recognized the puzzles were impossible to solve.

### Outcome variables of interest

#### Parenting and relationship self-report assessments

Here, closeness of the parent–child relationship was independently assessed in children and parents using a well-validated, single-item pictorial scale (Inclusion of Other in the Self Scale, IOS) (Aron et al., 1992), where higher scores represent increased closeness. To evaluate domains of parenting that have been previously related to mental health outcomes in children, we assessed self-reported parenting stress (Parenting Stress Scale, PSS; 18-items, 5-point Likert scale, higher scores representing increased parenting-related stress) (Berry and Jones, 1995), frequency of mindful parenting practices (Interpersonal Mindfulness in Parenting, IMP; 31-items, 5-point Likert scale, higher scores representing more frequent practice) (Duncan, 2007; Duncan, 2025), and the beliefs that caregivers hold related to their child’s emotional experience (e.g., the value of emotions, how emotions should be managed and expressed; Parents’ Beliefs about Children’s Emotions, PBACE) (Halberstadt et al., 2013). Total scores were calculated for each scale, except for the PBACE, where the seven validated subscales were used. The subscales quantified a caregivers’ belief that children’s positive emotions may be harmful (“Cost of Positivity”), children’s anger can be useful (“Value of Anger”), children use emotions to manipulate others (“Manipulation”), children can control their negative emotions (“Control”), the importance of parents being aware of their child’s emotions (“Parental Knowledge”), children can manage their feelings independently of their caregivers (“Autonomy”), and that children’s emotions are stable and long-lasting (“Stability”) (Halberstadt et al., 2013). Of note, this scale has been validated across three ethnic groups in caregivers of children aged 4–10 years old, thus, overlapping with our 8–11-year-old participants but slightly younger aged children. In general, caregivers who report high levels in the cost of positivity, manipulation, control, autonomy, and stability tend to report less supportive caregiving behaviors; conversely, caregivers who report high in the value of anger and knowledge subscales tend to report more supportive caregiving behaviors (Halberstadt et al., 2013). Finally, while change and assessment details are reported elsewhere (Lathren et al., 2026), we included the Self-Compassion Scale (SCS), both caregiver (SCS, 26-items, 5-point Likert scale) and child (SCS-Youth, 17-items, 5-point Likert scale) reports (Neff, 2016; Neff et al., 2020), where higher scores indicate more frequent practice of self-compassion, as a potential moderator as it is the primary target of the intervention.

#### Child emotional arousal during the dyadic stressor task

During the Performance Challenge Task, patterns of the child stress response were captured using two indirect measures of the sympathetic and parasympathetic nervous system function: autonomic skin conductance levels (SCL) (Critchley and Nagai, 2020) and heart rate variability (HRV) (Grégoire et al., 2023). Electrodermal activity (EDA) and electrocardiography (ECG) signals, for SCL and HRV analyses, respectively, were separately recorded at a sampling rate of 500 Hz using a MindWare Mobile device (MindWare Technologies LTD, Gahanna, OH). To quantify baseline levels of arousal, all physiological markers were also collected while watching a 5-min nature video prior to completing the task. EDA and ECG were investigated only within the unsolvable puzzle trials for each cohort.

Electrodermal activity (EDA) was recorded via two Ag/AgCl MindWare electrodes placed on the index and middle fingers of the participant’s left hand. Preprocessing was performed using the MindWare Technologies EDA applications software (v3.2.13). Trained students visually inspected each EDA file to manually remove abnormal peaks or valleys indicative of corrupted signals and the signal was subsequently smoothed using a rolling filter with a block size of 500 data points. The average skin conductance level (SCL) during each puzzle trial and during the baseline period, measured in MicroSiemans, served as a proxy for baseline and trial-level changes in arousal, respectively. Before statistical analysis, SCL values were *z*-scored, and their normality was confirmed using the Shapiro–Wilk test.

Electrocardiography (ECG) was collected using the three-electrode snap configuration (i.e., bilateral lower ribs and right clavicle). Preprocessing of the ECG signal was conducted using MindWare Technologies HRV applications software (v3.2.13). The software first performed automated R-peak detection (i.e., identifying heartbeats) and calculated the inter-beat interval (IBI) time series. Next, the time series was detrended to remove the global linear trend and filtered using a Hamming window function. Quality control involved trained personnel manually inspecting the data to remove obvious artifacts and confirm the accuracy of the automated beat detection (Berntson et al., 1997). Following standardized procedures when studying the stress response (Castaldo et al., 2015), the resulting R-R intervals were used to calculate the Root Mean Square of Successive Differences (RMSSD). This time-domain measure served as our primary indicator of parasympathetic nervous system activity (Kleiger et al., 2005). RMSSD was selected for its reliability and decreased sensitivity to respiratory rate as compared to other common HRV metrics (e.g., high frequency power) (Hill and Siebenbrock, 2009). RMSSD was calculated as the average during the baseline period and during each puzzle trial and *z*-scored. Normality was confirmed via the Shapiro-Wilkes test prior to statistical analysis.

#### Caregiver supportive and overcontrolling behavior during the dyadic stressor task

Caregivers and children were video recorded during the stressor task. Forty-two *v*ideos with both pre- and post-intervention videos were blinded such that subject ID and timepoint were hidden to coders. Two steps were taken to establish reliability: First, two trained coders worked with an expert coder and scale developer to rate two behavioral constructs (caregiver support and caregiver overcontrol) in 10 videos randomly selected (5 from each cohort) using the Behavioral Involvement Parenting Scale (BIPS) codebook (ICC(3) = 0.91 for support, ICC(3) = 0.94 for overcontrol) (Rasmussen and Borelli, 2015). After reliability was established with the expert coder, the two coders double-coded half of the videos (21 videos, 10 from cohort 1) to further confirm their reliability with each other (ICC(2) = 0.94 for support, ICC(2) = 0.96 for overcontrol). After reliability was established, the remaining 21 videos were divided amongst the two coders for scoring. For analysis, one coder’s ratings were randomly selected for each video that was double-coded.

The BIPS coding manual is based on established conceptualizations of supportive (e.g., warm, responsive, and autonomy-supportive behaviors) and overcontrolling (e.g., intrusive, insensitive, autonomy-restrictive behaviors) caregiver behaviors (Grusec and Davidov, 2007; Bögels and Brechman-Toussaint, 2006) and has been used in prior research (Borelli et al., 2018; Borelli et al., 2017) During each of the six unsolvable puzzle tasks, blinded coders rated caregiver behavior using the support scale (eight binary, yes/no items; sample item: “caregiver praises child success or partial success”) and the overcontrol scale (9 binary, yes/no items; sample item: “caregiver gives unsolicited verbal instructions”). See Supplementary Appendix C for a complete list of behaviors coded. Coders applied a score of “0” (indicating the absence of a particular behavior during each puzzle) or “1” (indicating the presence of that behavior at least once during each timed puzzle). Support and overcontrol scores were averaged across the puzzles for mean scores for each task timepoint for each dyad. Higher scores indicate higher levels of support and overcontrol.

#### Caregiver and child reflective functioning

After the task was completed, dyads were separately interviewed by trained research assistants using a standardized, structured set of questions used in previous studies and adapted from the Parent Development Interview—Revised: School-aged Children (Slade et al., 2016). First, to facilitate recalling their experience, participants each viewed a recording of the dyad during two puzzles from the task. Then, the research assistant asked four questions about the participant’s own thoughts and emotions during the task (to determine self-focused reflective functioning), and four questions about their guesses at their dyadic partner’s emotions and thoughts during the task (to determine child or parent-focused reflective functioning). A sample question is: “What do you think you/your child/your parent were/was thinking during the puzzles?” (see Supplementary Appendix B for a table of all questions). These interviews were transcribed verbatim and de-identified prior to coding.

Two trained coders (one expert coder) blinded to timepoint used a coding scheme grounded in Fonagy’s and Slade’s systems for coding reflective functioning (RF) (Fonagy et al., 1998) and adapted for use with shorter interviews (Borelli et al., 2017). This coding scheme has been validated in other studies (Borelli et al., 2017) and is based on participants’ use of mental state language (i.e., describing thoughts, emotions, wishes, or motivations) as well as their linking of these mental states to other mental states or behaviors. This process, called mentalizing, indicates a capacity to recognize and understand emotions in themselves and others (also called mentalizing) and to understand how emotions affect behavior.

Coders rated answers to each question on a scale from −1 (indicating active antagonizing of interviewer or distortion of thoughts or emotions) to 7 (indicating sophisticated levels of mentalizing as evidenced by multiple examples of linking mental states to behaviors or mental states to other mental states). A score of 1 indicates a non-response (e.g.,” I do not know”), a score of 2 indicates an answer that describes a behavior or trait instead of a mental state (e.g., “He did well on the puzzles”), and scores between 3 and 7 indicate mental state(s) are mentioned or linked with behaviors with increasing frequency and clarity (e.g., “He likely felt embarrassed about his performance [mental state] even though he kept saying he did not care about how he did [behavior]”). A score of 5 indicates a basic level of mentalizing. Other indicators of a score of 5 for mentalizing, in addition to linking with behavior, include the recognition that people can hide emotions, that children’s ability to express their mental states is age-dependent, and the capacity to identify emotions underlying a behavior.

Composite RF scores for child and caregiver were the final dependent variable in statistical analyses. These composite scores were created by first computing the average of the two coders’ ratings for each question asked in the interview, split into self-focused or other-focused RF. There were four questions each for self- and other-focused RF in caregivers and children (except child self-focused RF, which had only three questions). The overall composite RF score was an average of the self- and other-focused RF for child and caregiver. Raters achieved reliability on both the child, ICC(2) = 0.86 and the caregiver RF, ICC(2) = 0.89. Due to variation in interviewers, some caregivers answered less than 4 self-focused questions (on average, 3.6 questions were asked) and child-focused questions (on average, 3 questions were asked). For children, an average of 3.7 self-focused questions were asked and 2.7 caregiver-focused questions were asked. Self- and other-focused scores were calculated as the average, rather than the sum of questions that were administered, meaning that interviews with fewer questions may carry somewhat greater measurement error.

### Statistical analyses

#### Significance of change between pre- and post-intervention

All statistical analyses were completed using R (v4.5.1) and RStudio (v2025.09.1 + 401). Due to similarity of baseline demographic and symptom variables (Lathren et al., 2026), responses from both cohorts were consolidated for analyses to increase power and sample size. First, changes from baseline to post-intervention in the constructs of parenting values and stress, caregiver task behavior, and reflective functioning were assessed. A conditional testing strategy was employed to assess intervention-related change in outcome measures. Here, a Shapiro-Wilkes Test (SWT) was first run on the difference scores (post-intervention minus pre-intervention) to assess normality. A non-significant SWT suggests a normal distribution and resulted in a parametric paired t-test, while a significant SWT suggests the absence of normality and resulted in a non-parametric paired-sample Wilcoxon signed-rank test. The appropriate test statistic (*t* or *V*) and the corresponding *p*-value for all analyses are reported. For all paired *t*-tests, the *t*-statistic reflects the sign of the pre- minus post-intervention difference (i.e., a negative *t*-statistic therefore indicates an increase from pre- to post-intervention, and vice versa). Note that the *V*-statistic from the Wilcoxon test, which represents the sum of positive ranks, should not be used to interpret the magnitude or compare differences across outcomes. Within any outcomes exhibiting significant pre-post change, an exploratory change-score correlations (Pearson’s r correlation) was run with all other change scores. For any significant change detected, we calculated the standardized effect size with 95% confidence intervals using the effectsize package in R (Cohens *dz* for significant parametric tests; matched-pairs rank-biserial *r* for significant non-parametric tests). The effect size estimate will reflect the sign of pre- minus post-intervention difference, so a negative effect size estimate reflects an increase from pre- to post-intervention and vice versa.

Due to the increased power of task-specific physiological measures (i.e., SCL, HRV), time-related changes in child stress response were examined next. Models failed the assumption of the homogeneity of variance (variances within each session are statistically unequal) and rendered running an ANCOVA inappropriate. Therefore, conservative yet powerful linear mixed-effects models were used. Here, the interaction of Trial (1–6) and Session (Pre- and Post-Intervention) on average SCL and RMSSD were estimated, controlling for age, sex assigned at birth, average baseline physiology, cohort, and subject as a random-effect. Cohort was included given the change to the Performance Challenge Task following Cohort 1, as described above, such that pooled-cohort models account for the resulting difference in task conditions between cohorts. Three child participants refused ECG data collection and were therefore left out of subsequent HRV statistical models. If a physiological measure (SCL, RMSSD) exhibited a significant time-related change, the presence of baseline cohort moderators was then investigated.

#### Baseline dyadic characteristics as moderators of change

Finally, and examination of whether any dyadic characteristics were predictive of experiencing the largest (or smallest) benefit of the MSC-CC intervention was performed, as quantified by change in child stress response. A series of exploratory moderation analyses were separately run for each of the following potential baseline moderators: mindfulness in parenting (IMP), self-compassion skills (SCS; child and caregiver), parenting stress (PSS), parent–child closeness (IOS, child and caregiver), parents’ beliefs about their child’s emotions (PBACE subscales), and reflective functioning (child and caregiver). Each model included an interactive term between session (pre, post) and the potential moderator. This approach was chosen because each moderator may relate to the physiological stress response through a distinct mechanism and because it is the standard practice in small, exploratory pilot studies, where testing multiple predictors simultaneously in one model is often infeasible given limited power (Leon et al., 2011). As in the primary SCL/RMSSD model above, each moderator model included subject as a random effect to account for the non-independence of repeated puzzle trial observations within participants. Follow-up regressions were run on any models that detected moderation to probe the significance of simple slopes and visualize using split-mean moderators (high vs. low levels of the moderator at baseline).

Across all primary and moderation analyses, degrees of freedom for linear mixed-effects models were Satterthwaite-approximated using the lmerTest package in R. Further, given the pilot, hypothesis-generating nature of this study and the resulting risk of reduced power with any correction, no adjustment for multiple comparisons for pre-post outcome changes or moderator analyses. Findings should therefore be interpreted as preliminary rather than confirmatory.

## Results

### Cohort characteristics

Dyads consisted of young children (*M* = 8.72 years, range = 8–10; Male, *n* = 12; Female, *n* = 11) and a primary caregiver (*M* = 41.26, range – 31–50; Mother, *n* = 20; Father, *n* = 3). Families were predominantly white (Child, *n* = 18; Caregiver, *n* = 20) and generally high socioeconomic status across income ($100 k + annual income, *n* = 18) and highest level of parental education (College degree or higher, *n* = 18). Correlations between study variables, including parenting beliefs and relationships, can be found in Table 1 and further demographic breakdowns are published elsewhere (Lathren et al., 2026).

**Table 1:** Correlations between parenting, values, and behavior.

Questionnaire	**Parent–Child Closeness (Caregiver)**	**Parent–Child Closeness (Child)**	**Parenting Stress**	**Cost of Positivity (PBACE)**	**Value of Anger (PBACE)**	**Manipulation (PBACE)**	**Control (PBACE)**	**Parental Knowledge (PBACE)**	**Autonomy (PBACE)**	**Stability (PBACE)**	**Mindfulness in Parenting**	**Caregiver Support (PCT)**	**Caregiver Overcontrol (PCT)**	**Reflective Functioning (Child)**	**Reflective Functioning (Caregiver)**	**Self-Compassion (Caregiver)**	**Self-Compassion (Child)**
Parent–child closeness (Caregiver)	1																
Parent–child closeness (Child)	0.020	1															
Parenting stress	−0.102	−0.161	1														
Cost of positivity (PBACE)	−0.057	−0.150	0.348*	1													
Value of anger (PBACE)	−0.141	0.000	−0.117	0.167	1												
Manipulation (PBACE)	0.019	−0.077	0.522**	0.563**	0.214	1											
Control (PBACE)	0.132	−0.070	0.059	0.204	−0.028	0.271	1										
Parental knowledge (PBACE)	0.282	−0.096	0.394*	0.100	−0.003	0.222	−0.292	1									
Autonomy (PBACE)	0.236	−0.151	0.179	0.126	−0.052	0.325*	0.299	−0.077	1								
Stability (PBACE)	0.527***	0.204	−0.081	0.091	−0.098	−0.121	0.034	0.399*	0.044	1							
Mindfulness in parenting	0.036	0.044	−0.578***	−0.157	−0.073	−0.403*	0.080	−0.487*	−0.028	−0.133	1						
Caregiver support (PCT)	−0.062	−0.158	0.244	0.294	−0.113	0.228	0.125	0.063	−0.030	−0.095	0.175	1					
Caregiver overcontrol (PCT)	0.070	−0.049	0.449*	0.344*	−0.035	0.631***	−0.011	0.537**	0.212	−0.004	−0.239	0.247	1				
Reflective Functioning (Child)	−0.313*	0.177	−0.156	0.188	0.338*	0.055	0.135	−0.109	−0.134	0.077	−0.091	−0.055	−0.041	1			
Reflective functioning (Caregiver)	−0.206	−0.066	−0.022	−0.008	0.000	−0.084	0.280	−0.305*	−0.240	−0.129	−0.040	0.144	−0.342*	0.179	1		
Self-compassion (Caregiver)	0.01	0.11	−0.36*	−0.01	−0.18	−0.29	0.13	−0.39*	−0.22	−0.08	0.76***	0.35*	−0.18	−0.12	0.20	1	
Self-compassion (Child)	0.18	0.17	−0.28	−0.45*	0.06	−0.17	−0.15	−0.13	0.03	0.08	−0.08	−0.30	−0.14	−0.18	−0.15	−0.16	1

Each box reflects the *r*^2^ value for each correlation. Significance is denoted by asterisks: *** (*p* < 0.00001); ** (*p* < 0.001); * (*p* < 0.05). PBACE, Parental Beliefs about Child’s Emotions; PCT, Performance Challenge Task.

### Time-related changes in dyadic relationship and parenting practices

Descriptive statistics and results of parametric and non-parametric approaches can be found in Table 2. We detected significant changes after the MSC-CC dyadic intervention in four key outcomes: mindfulness in parenting (*V* = 53.5, *p* = 0.047, *r* = −0.490, 95% CI [−0.772, −0.048]), caregiver-reported parent–child closeness (*V* = 86, *p* = 0.046, *r* = 0.467, 95% CI [0.018, 0.759]), and caregiver’s belief about the value of anger (*t*(20) = −2.86, *p* = 0.009, *dz* = −0.640, 95% CI [−1.114, −0.153]) and caregiver’s belief that their child can manage emotions independently (*t*(20) = −2.40, *p* = 0.026, *dz* = −0.538, 95% CI [−1.00, −0.063]). All intervention-related changes are visualized in Figure 2A. Interestingly, caregiver-reports of closeness with their child *decreased* from pre- to post-intervention; this change correlated with a decrease in parenting stress (*r* = −0.595, *p* = 0.009; Figure 2B). In contrast, as hypothesized, we saw significant increases in the utilization of mindfulness in daily parenting practices. Outside of *a priori* hypotheses, we also observed increases in the caregiver’s beliefs that anger can be a useful emotion and that children can independently manage their own emotions. The positive change in the value of anger was further correlated with decreased parenting stress (*r* = −0.622, *p* = 0.006; Figure 2C) and increased behavioral support during the dyadic repeated stressor task (*r* = 0.473, *p* = 0.048; Figure 2C). Finally, we did not detect significant changes in caregiver stress, caregiver behavior, or caregiver/child total reflective functioning scores.

**Table 2:** Descriptive statistics and significance of change for key dyadic and parenting outcomes.

Questionnaire	**Average**		**Shapiro-Wilkes normality**		**Test** **performed**	**Significance OF Change**	
	Pre-intervention	Post-intervention	*W*	*p*		Test statistic	*p*
Dyadic relationship and stress							
Parent–child closeness (Caregiver)	4.667	4.333	0.750	0.000	Non-Parametric	88.000	0.046*
Parent–child closeness (Child)	5.381	6.000	0.860	0.006	Non-Parametric	14.500	0.103†
Parenting stress	43.095	40.048	0.840	0.003	Non-Parametric	161.000	0.116
Parenting beliefs and values							
Cost of positivity (PBACE)	8.524	8.048	0.921	0.089	Parametric	0.681	0.504
Value of anger (PBACE)	23.714	25.619	0.966	0.649	Parametric	−2.864	0.009*
Manipulation (PBACE)	8.048	8.571	0.947	0.294	Parametric	−0.959	0.349
Control (PBACE)	12.667	12.810	0.967	0.668	Parametric	−0.174	0.864
Parental knowledge (PBACE)	13.143	12.000	0.964	0.594	Parametric	1.975	0.062†
Autonomy (PBACE)	17.571	19.429	0.940	0.218	Parametric	−2.404	0.026*
Stability (PBACE)	12.524	12.524	0.966	0.646	Parametric	0.000	1.000
Mindfulness in parenting	101.286	106.667	0.817	0.001	Non-Parametric	53.500	0.047*
Caregiver behavior during the dyadic stressor task							
Caregiver support (PCT)	15.476	15.905	0.962	0.568	Parametric	−0.299	0.768
Caregiver overcontrol (PCT)	6.619	8.571	0.886	0.019	Non-Parametric	36.000	0.180
Reflective functioning							
Reflective functioning (Child)	2.740	2.620	0.855	0.005	Non-Parametric	135.50	0.498
Reflective functioning (Caregiver)	4.080	4.054	0.970	0.734	Parametric	0.168	0.869

Normality was assessed using the Shapiro-Wilkes test of normality where a non-significant p-value suggests normality. Pre-post changes across all domains reports differences in pre- and post-intervention assessments using parametric (paired *t*-test, t) and non-parametric methods (paired-sample Wilcoxon signed-rank test, V), where appropriate. Direction and magnitude of differences cannot be interpreted from the *V*-test statistic. Significance is denoted by the *p*-value: ** (significant), † (trending). PBACE, Parental Beliefs about Child’s Emotions.

**Figure 2:**
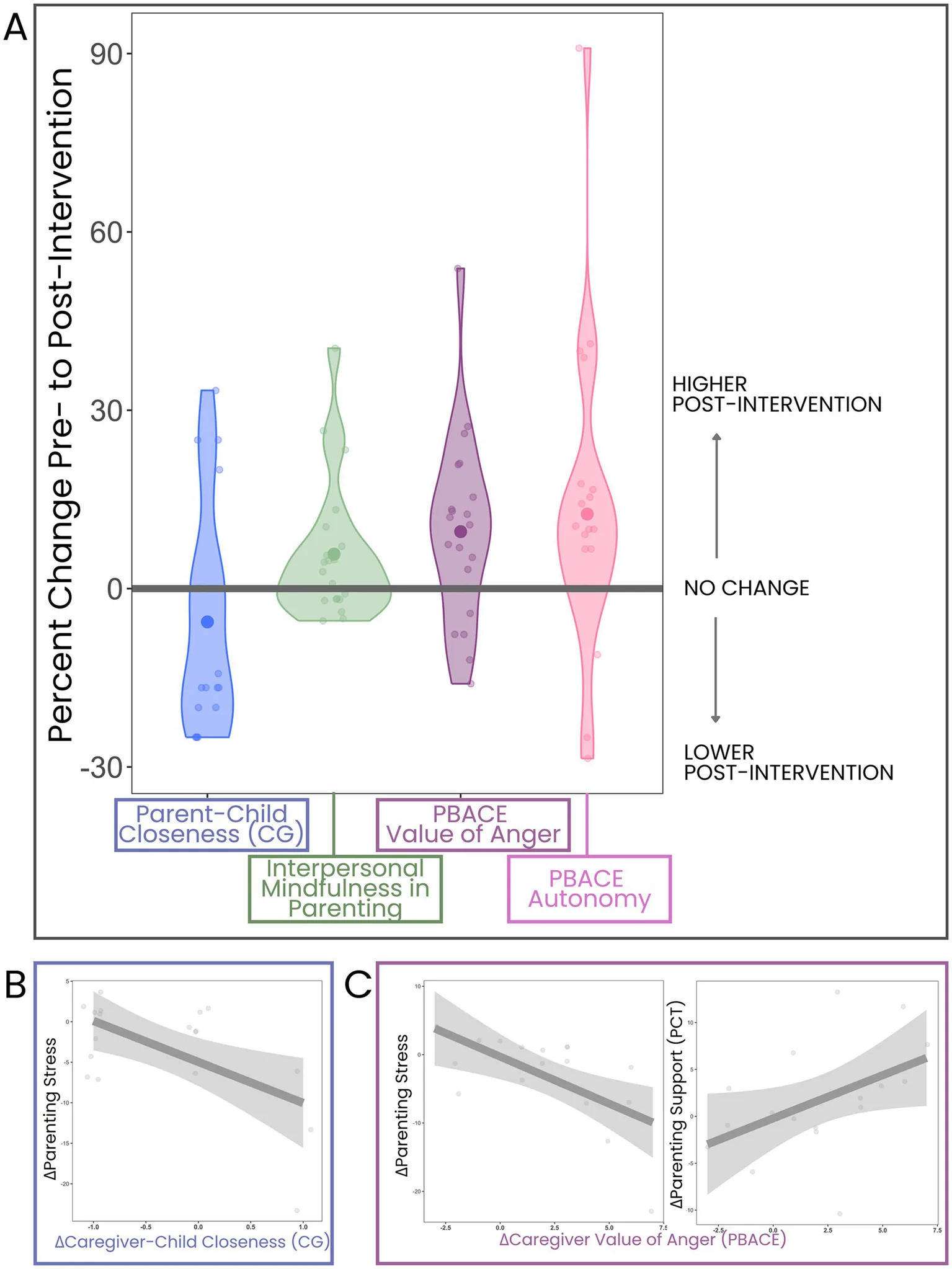
Pre-to-post intervention changes in parenting practices and relational outcomes. **(A)** Violin plots depicting pre- to post-MSC-CC changes in the four self-report measures that showed significant change: caregiver-reported parent–child closeness (IOS, blue), Interpersonal Mindfulness in Parenting (IMP, green), caregiver belief in the value of children’s anger (PBACE-Value of Anger, purple), and caregiver endorsement of children’s emotional autonomy (PBACE-Autonomy, pink). Each dot represents an individual participant; the violin plots display the distribution and central tendency. **(B)** Scatter plot depicting the significant positive correlation between change in caregiver-reported parent–child closeness (ΔIOS) and change in parenting stress (ΔPSS). **(C)** Scatter plots depicting the significant correlations between change in caregiver belief in the value of anger (ΔPBACE-Anger Value) and change in parenting stress (ΔPSS; left panel) and change in observed caregiver behavioral support during the dyadic stressor task (right panel).

### Baseline dyadic characteristics predicting change in the child stress response

First, we investigated physiological changes after MSC-CC participation. Skin conductance level (SCL) indexes sympathetic nervous system activation, with lower SCL reflecting lower physiological arousal. Linear mixed-effects models detected no interactions between session (pre, post) and puzzle trial number in SCL [*t*(267) = 1.59, *p* = 0.113] or RMSSD [*t*(191) = 1.50, *p* = 0.165]. However, there was a significant main effect of session (pre, post) in SCL [*t*(268) = 2.43, *p* = 0.016], controlling for baseline physiology, age, sex, and cohort number. Across all puzzle trials, we see significant decreases in average SCL from pre- to post-intervention (Figure 3A). Sensitivity analyses ran this model separately within each cohort. Consistent with expected reductions in power when splitting a small sample, the main effect of session was not significant in either cohort (*p*’s > 0.05).

**Figure 3:**
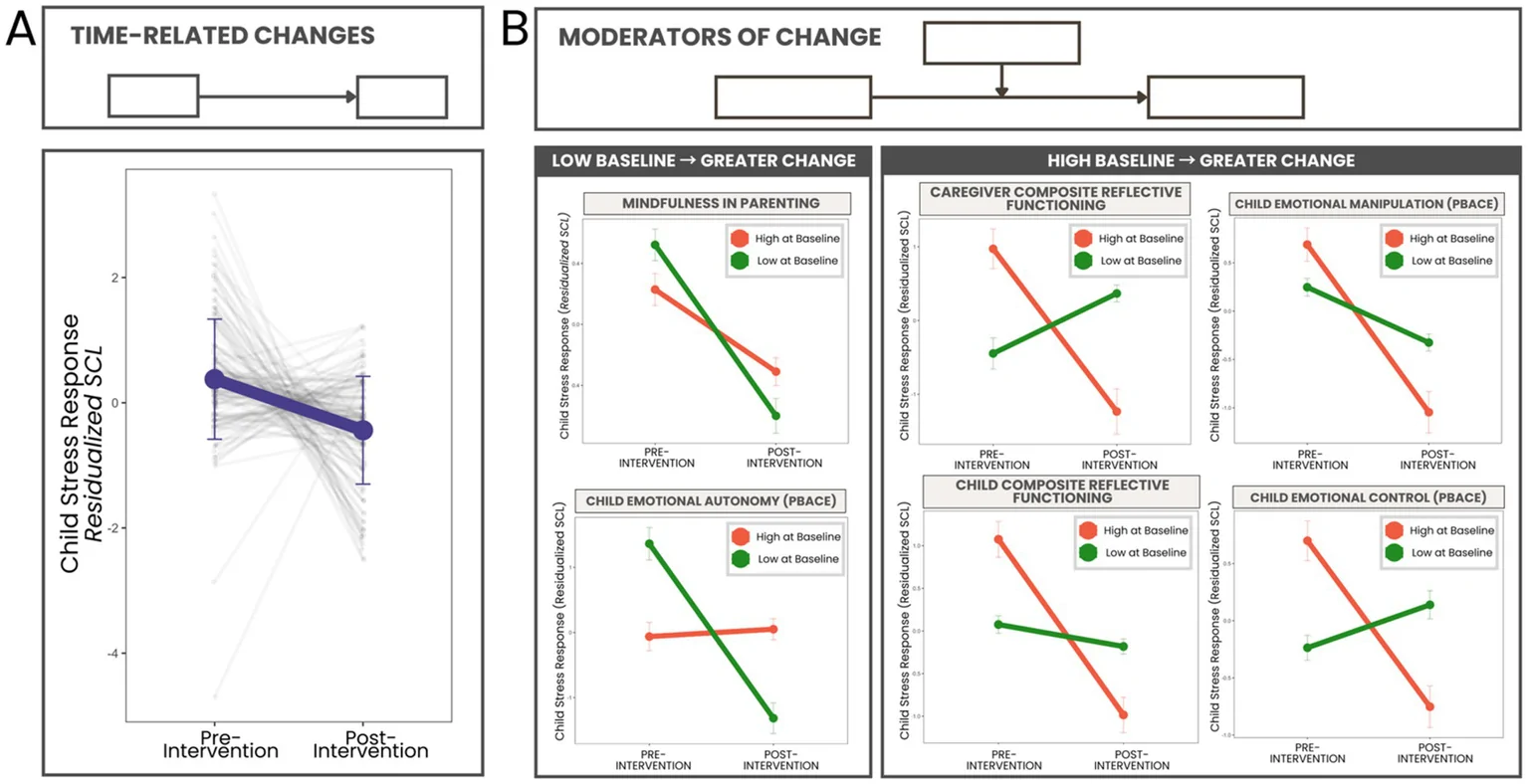
Child physiological stress response changes and baseline moderators following MSC-CC participation. **(A)** Line graph depicting average *z*-scored skin conductance level (SCL; μS) across six unsolvable puzzle trials at pre- and post-intervention assessment. A significant main effect of session was detected, reflecting an overall reduction in SCL from pre- to post-intervention across all puzzle trials. Gray lines represent individual participant during one puzzle and the bold purple line represents the overall group average. **(B)** Line graphs of significant moderating relationships, depicting average SCL across all puzzles pre- and post-intervention. Slope of change is depicted by the level of a specific baseline characteristic (green, low at baseline; red, high at baseline). In the left box, variables associated with the greatest reduction in child stress among dyads with low baseline levels include: mindfulness in parenting (IMP, left-top) and children’s emotional autonomy (PBACE-Autonomy, left-bottom). In the right box, variables associated with the greatest reduction in child stress among dyads with high baseline levels include: caregiver reflective functioning (left-top), child reflective functioning (left-bottom), belief that children use emotions to manipulate (PBACE-Manipulation, right-top), and belief that children can control their emotions (PBACE-Control, right-bottom).

We then assessed whether dyadic or parenting characteristics at study enrollment show significantly different impacts of the intervention on child stress response. Interestingly, many dyadic characteristics assessed in this study were significant moderators of the reduction in child physiology following MSC-CC (Figure 3B). First, dyads that entered the study with *low levels* of the following variables showed the *greatest* reduction of child stress during the dyadic repeated stressor task: mindfulness in parenting practices [IMP, *t*(280) = −2.91, *p* = 0.004], child self-compassion skills [SCS-Y, *t*(277) = −2.71, *p* = 0.007], caregiver reports of caregiver-child closeness [IOS, *t*(272) = 2.52, *p* = 0.012], and endorsement that children can manage emotions independently [PBACE-Autonomy, *t*(272) = −2.92, *p* = 0.004].

On the other hand, dyads that entered the study with *high levels* of the following variables showed the *greatest* reduction of child stress during the dyadic repeated stressor task: overcontrolling behavior [PCT Overcontrol, *t*(261) = 3.90, *p* < 0.001], higher child reports of parent–child closeness [IOS-Child, *t*(283) = −3.69, *p* < 0.001], higher reflective functioning in both children [*t*(276) = 8.73, *p* < 0.001] and parents [*t*(279) = 7.56, *p* < 0.001], stronger beliefs that children use their emotions to manipulate [PBACE-Manipulation, *t*(282) = 4.54, *p* < 0.001] and that children can control their emotions [PBACE-Control, *t*(277) = 5.40, *p* < 0.001].

## Discussion

This pilot study investigated exploratory impacts of Mindfulness and Self-Compassion for Children and Caregivers (MSC-CC), an accessible and virtual dyadic intervention, on parenting and child stress physiology during a dyadic stressor task. MSC-CC is a dyadic program providing families with the age-appropriate tools that they need to promote health and well-being of both children and parents (Lathren et al., 2026). As this investigation recruited a small cohort of families with younger children, we implemented a classically-designed stressor task that would allow us to physiologically measure a child’s response to a challenging dyadic environment in a pre-post design (Borelli et al., 2018). This task provides a robust measure of ANS dysregulation, a known contributor to the development and maintenance of psychopathology (Young-Southward et al., 2020; de Kloet et al., 2005), thereby facilitating insight into the intervention’s psychobiological impact while effectively mitigating difficulties related to reporter bias and low statistical power. Our initial pilot study, which did not include any comparison condition, reports that this program may not only be associated with favorable changes in self-reported mindful parenting practices and emotion-related beliefs but also a significant reduction in the child’s stress response (indexed by SCL, not significant using HRV) during an emotionally demanding activity. Baseline moderators of ANS dampening reveal that dyads with bigger gaps in emotion-supportive parenting, controlling behavior, and less frequent practice of mindfulness in parenting show the greatest reductions in child stress. While preliminary and unable to rule out the possibility of time- or practice-related effects, these findings may help to identify dyads that may maximally benefit from this intervention and will be instrumental in shaping the protocol of a well-powered randomized controlled trial to evaluate intervention efficacy.

In addition to our previously-reported reductions in caregiver-reported child anxiety and depression symptoms and increases in child self-compassion skills following MSC-CC (Lathren et al., 2026), we now report significant and positive changes in self-reported mindful parenting practices, a finding aligned with a previous study (Lathren et al., 2023), as well as two emotion-related beliefs: that there is value in children feeling anger and that children can manage their difficult emotions independently. To our knowledge, this is the first study to examine changes in parent emotion-related beliefs associated with self-compassion intervention. Other research suggests caregivers who endorse valuing and understanding anger as a valid emotion (and a possible indicator of deeper, more vulnerable emotions) tend to have higher capacity to express their own negative emotions and to support their children’s expression of negative emotions (Halberstadt et al., 2013). It is thought that this belief helps caregivers of pre-adolescent children to accept and provide support for their child to express their anger in a clear and productive manner rather than dismissing, rejecting, or invalidating their anger, helping children to recover more quickly (Rogers et al., 2016). Furthermore, in this sample, greater valuing of children’s anger correlated with decreased parenting stress and increased behavioral support during stressful activities. This raises the possibility that increases in caregivers’ belief that children’s anger is valuable may be associated with adaptive changes in caregiver behaviors.

Meanwhile, endorsing the belief that children can manage emotions independently has been linked to *less* supportive caregiving behaviors in caregivers of children ages 4–10, potentially indicating a dismissive approach towards their distressed child (Halberstadt et al., 2013). Some of these findings may require a nuanced view. For example, as children approach adolescence, evidence suggests caregivers increase their expectations that children will manage their own emotions (Stettler and Katz, 2014), expecting emotional maturity may lead to less sensitive caregiving responses. However, other research contends that the benefits of providing specific types of emotional support that are age and child-specific, and that believing in the child’s growing capacities for self-regulation and providing less or different types of emotional support can be an important indicator of age-appropriate adjustment (Castro et al., 2018; Raeymaecker et al., 2022). In the context of this intervention, increases in this belief may be explained by both the age of the children and the content of the intervention. Given this was a dyadic intervention where pre-adolescent children were learning skills to be *self*-supportive towards their difficult emotions, one possible explanation is that caregiver participants grew in their beliefs that recognize their child’s capacity to manage emotions autonomously as a reflection of the child’s genuine skill-building during the course. Likewise, considering caregivers in the current study simultaneously grew in mindful parenting practices (i.e., practices that are emotionally attuned and responsive), this may suggest that this increased belief in their child’s emotional autonomy is adaptive and signals growth in their child’s self-regulation skills rather than a sign of disengagement or emotional dismissiveness. However, given the nuances of emotional support in this age range, this finding remains ambiguous. We cannot rule out that this finding instead replicates a previous association between autonomy and less emotionally supportive caregiving (Halberstadt et al., 2013). Therefore, further exploration of the impact of this change in belief post-intervention is warranted before drawing firm conclusions.

Unexpectedly, we also found a significant *decrease* in caregivers report of relational closeness with their participating child post-intervention. We used a well-validated and commonly used visual scale of overlapping circles (the Inclusion of Self in Other scale, or IOS) (Aron et al., 1992), which has been found to correlate positively with other relationship closeness measures and is thought to tap into one’s sense of interconnection with others in cross-sectional or observational studies. However, there is a dearth of evidence for this scale being used in an interventional setting. Interestingly, the children reported non-significant increased relational closeness with their caregiver pre versus post intervention using the IOS scale, and qualitative interview data with caregiver participants suggests increased closeness, connection and authenticity with their child (Lathren et al., 2026). Given these discrepancies, it is plausible that the graphic was interpreted differently by these caregivers, who also reported significant changes in their beliefs that children can manage their emotions autonomously. One plausible explanation is that that MSC-CC participation helped to foster a more balanced sense of individuation and boundary setting during this pivotal period of developmental change for the child, which may be reflective of lower scores of physical closeness with their child that may still be consistent with a healthy parent–child relationship. This aligns with prevailing theories of attachment and autonomy as children approach adolescence, which suggest secure attachment shifts from physical presence and safety to “felt security.” (McElhaney et al., 2009) However, as this is speculative and was not directly assessed, future studies could investigate whether this decrease was indeed beneficial and include other measures of relational closeness as well as a measure of caregivers’ perception of their child’s emotion regulation skills (Freitag et al., 2023). Inclusion of these additional measures could help tease apart these issues in the context of a dyadic intervention where children are gaining emotional skills.

Next, consistent with our hypotheses, we observed a significant reduction in the child stress response pre- to post-intervention, defined as average SCL during unsolvable puzzles. As a branch of the autonomic nervous system, the sympathetic nervous system mobilizes the available resources to respond to the current environmental demands (Porges, 2003) and its activation can be indirectly measured by skin conductance signals (Amin and Faghih, 2021). This finding indicates lower task-related physiological arousal following the intervention and does not, on its own, demonstrate improved or more adaptive stress regulation. The adaptive calibration model (ACM) of stress responsivity posits that individual differences in stress responsivity are the result of an evolutionarily adaptive calibration of the biological stress systems to the quality of the surrounding environment (Del Giudice et al., 2011). Here, while speculative and unconfirmed with our trial design, high stress reactivity pre-intervention may reflect an adaptive, but high-cost, calibration to a perceived unsupportive or demanding environment while the time-related reductions may be due to a shift in the baseline set-point of the child’s stress response (e.g., a less reactive, lower-cost setting). Further, this reduction was independent of change across individual trials, meaning that stress did not compound nor decelerate between the first and last puzzles. While it cannot rule out expectancy effects due to the prior experience with the task, these findings may argue the children did not just learn to manage the puzzle sequence better or habituate to the stress, rather it was a systemic, tonic change in the overall arousal level that is consistent with the ACM’s prediction that stress responsivity is plastic and calibrated by the quality of the environment (Del Giudice et al., 2011).

Mechanistically, we speculate that perhaps MSC-CC helped children to reduce the tendency to self-criticize or catastrophize failure, particularly in situations where their parent is available for support. Consistent with a recent study in young adults linking self-compassion training to changes in physiology and mental health (Halamová et al., 2019), by fostering self-kindness, the intervention may lower the self-imposed threat level, reduce the physiological set-point, and therefore decrease long-term sensitivity to stress and psychopathology. Larger, well-controlled studies are needed to confirm whether these time-related changes in physiology are indeed mechanisms of therapeutic change, particularly in naturalistic and dyadic contexts outside of the program setting. While the lack of change in heart rate variability between pre- and post-intervention might suggest that the effect was primarily on sympathetic tone rather than vagal responsivity (Young-Southward et al., 2020), it may also simply reflect inadequate power due to ECG data loss from the current cohort. However, future studies with higher ECG collection compliance are needed to draw firm conclusions.

Finally, although exploratory in nature, we examined moderators of the change in child stress physiology with the aim of understanding which dyads may maximally benefit from MSC-CC, where results represent exploratory patterns or candidate processes rather than demonstrated mechanisms of change. Caregivers starting with lower levels of potentially adaptive characteristics upon study enrollment (e.g., low mindfulness in parenting, low caregiver-perceived parent–child closeness, low belief that children can be autonomous in their emotion regulation) showed the greatest reduction in child arousal. In contrast, caregivers starting with higher levels of potentially maladaptive characteristics (e.g., higher levels of caregiver overcontrolling behavior during the dyadic stress task, higher levels of beliefs that children use emotions to manipulate and that children can control their emotions) also showed the most significant reductions in child arousal. While speculative, one possible explanation is that MSC-CC may be providing certain dyads with new coping and relational strategies that translate to greater benefit in the child’s stress response, where dyads that had already implemented mindfulness in their daily parenting practice or had more adaptive emotion-related beliefs prior to participating in this intervention may have had less room for improvement (i.e., ceiling effect). Further, it is also possible that caregivers who show overcontrolling behavior or have beliefs that children use emotions to manipulate others may have high anxiety or distress themselves (Borelli et al., 2014a) and that participation may be associate with changes in caregiving patterns in ways that benefited their child’s stress response. However, given the small, exploratory nature of this analysis, these patterns should be considered candidate hypotheses for replication in future, adequately powered studies rather than established explanations. Given behavior is bidirectional, with the child’s behavior influencing the caregiver and vice versa, future work should take child behavior into account as an important influence on caregiver behavior and functioning.

Similarly, the fact that high baseline RF in both child and caregivers was a moderator suggests that those with a stronger pre-existing capacity for understanding nuanced connections between mental states and behaviors in themselves and others may be better equipped to translate the course’s conceptual learning into physiological change. Interestingly, this analysis also demonstrates that children with high levels of baseline RF experienced *higher* physiological stress at baseline compared to children low in this capacity; children whose caregivers were high in RF also experienced *higher* baseline stress. This runs contrary to previous empirical and theoretical research that indicates that caregiver RF is primarily a resource that is linked to secure attachment relationships, sensitive caregiving behaviors and positive child outcomes (Borelli et al., 2021; Luyten et al., 2017). However, it is plausible that caregivers who are high in child-focused RF could unintentionally cause more stress for their child by being hyperaware of their feelings or calling attention to behavioral cues that make the child more self-conscious—a finding that has been termed ‘intrusive mentalizing’ (Luyten et al., 2017). Moreover, RF capacities in middle childhood are not well studied; there may be nuanced interactions with other variables, like gender and age, that will be important to explore in future research. Thus, these data provide a foundation for further inquiry.

Despite the moderator role of behavior and RF on child stress physiology, contrary to our predictions, we did not detect any significant changes in caregiver observed behavior (increased supportiveness, decreased overcontrol) or caregiver/child RF associated with participation in MSC-CC. This may be due to low power in this small sample. Past research has suggested caregivers high in self-compassion respond more sensitively to their child (Psychogiou et al., 2016); and that after participating in caregiver-only compassion-based training, caregivers demonstrated lower hostility and verbosity when interacting with their child, which is conceptually similar to overcontrol (Kirby et al., 2023). Theoretically, self-compassion skills could help caregivers notice their own distress cues and support themselves, leading to lower levels of overcontrolling behavior in the context of the stress task. Likewise, MSC-CC provides language for supporting children compassionately during emotional challenges. Finally, given MSC-CC includes skills for greater emotional awareness, understanding and acceptance, it is plausible that both caregiver and child would grow in RF capacities; indeed, post-intervention qualitative data suggested some caregivers became more curious about and invested in understanding their child’s emotional states (Lathren et al., 2026). Thus, these measures are important to continue to include in future studies.

While this pilot study of MSC-CC is a critical step forward for dyadic self-compassion programming and potential biological mechanisms change, there are limitations that are important to consider. The small sample size with no comparison group restricts our ability to make any definitive statements on efficacy or mechanisms. This limitation is illustrated by the non-significance of session effects on SCL when run separately within each cohort, likely reflecting the reduced power of a split sample. Any changes detected here, while conservative and targeted, may be due to time, and experience with the puzzle task, or recognizing that the puzzles were solvable, rather than MSC-CC programming. Our team learned that adding puzzle task instructions to minimize caregiver help, using shortened puzzle task timing to limit children discovering the puzzles are unsolvable, and ensuring all questions are asked during the post-task interview may be important improvements to study administration. Additionally, the generally high socioeconomic status of the participants limits broader generalizability. While the required elevated symptoms in one cohort ensures the sample was in-part clinically relevant, it does not imply benefit for children diagnosed with internalizing disorders. Furthermore, the reliance on self-report and the non-specific physiological change (SCL) necessitates further investigation into behavioral and clinical outcomes. Future studies will include a definitive randomized controlled trial, a more diverse sample, and an examination of long-term outcomes to confirm these preliminary findings and further explore the mechanisms of observed benefit.

Despite these limitations, dyadic MSC-CC participation in pre-adolescence is associated with promising, accessible changes in caregiver-child relationships, stress responses, and emotion-related beliefs, skills, and interpersonal behaviors, prior to the emotionally tumultuous years of adolescence. The moderation effects noted suggest that the intervention’s mechanism of change is complex and likely operates through different pathways depending on the dyad’s initial characteristics, with specific baseline vulnerabilities or strengths associated with differential patterns of change in child stress physiology. Altogether, these findings lend additional evidence to prevailing models that mindfulness- and self-compassion informed practices, practiced in relationship, are useful in cultivating healthy emotional functioning in family relationships.

## Data Availability

The datasets presented in this article are not readily available because data contains sensitive health information and will not be made publicly available but may be available upon reasonable request. Requests to access the datasets should be directed to Sara Heyn, sheyn@mcw.edu.
