# Supplementary material for: Examining child stress response, parenting and relationship-related changes associated with a dyadic mindfulness and self-compassion program
Source: Front Psychol. 2026 Oct 15;17:1933287. doi: 10.3389/fpsyg.2026.1933287 (PMC13716759; doi:10.3389/fpsyg.2026.1933287)
Supplement: Supplementary file 1 [file Supplementary_file_1.DOCX]

**Supplementary Materials**

*(To be included as Supplementary Material for Online Publication)*

**Manuscript Title:** Examining Child Stress Response, Parenting and Relationship-Related Changes Associated with a Dyadic Mindfulness and Self-Compassion Program

**Authors:** Christine R. Lathren, MD, MSPH^1^, Jessica L. Borelli, PhD^2^, Rosemary Ellsworth^1^, Elondra Harr, MPH^3^, Samantha DiMaio, BA^4^, Jamie Lynn Tatera, MS^5^, Veronica Diaz, BS^4^, Karen Bluth, PhD^6^, Ryan J. Herringa, MD, PhD*^2^, Sara A. Heyn, JD, PhD*^2^

* Indicates co-senior authorship

^1^Department of Physical Medicine and Rehabilitation, University of North Carolina at Chapel Hill, CB 7200, Chapel Hill, NC 27599, USA

^2^Department of Psychology and Social Behavior, University of California, Irvine, 4201 Social & Behavioral Sciences Gateway, Irvine, CA, USA

^3^Department of Public Health Education, University of North Carolina at Greensboro, Greensboro, NC USA

^4^Department of Psychiatry, University of Wisconsin School of Medicine & Public Health; Madison, WI, USA

^5^Wholly Mindful, Milwaukee, WI, USA

^6^Department of Psychiatry, University of North Carolina at Chapel Hill, Chapel Hill, NC USA

| **Supplemental Table 1: Summary of MSC-CC Course Weekly Sessions** | | |
| --- | --- | --- |
| **Week** | **Session Name** | **Description and Sample Exercises** |
| 1 | Self-Compassion and Universal Emotions | Participants are introduced to each other and the course. They learn the 3 parts of self-compassion: (1) mindfulness represented by Spots the giraffe, (2) common humanity represented by Buddy the dog, and (3) self-kindness represented by Super Snuggles the bunny. Participants explore accepting *all* emotions through a Share the Plate exercise. |
| 2 | Mindfulness, Resistance and Common Humanity | Participants learn about noticing emotions, why we resist emotions, opening slowly to difficult emotions and why naming emotions is helpful (Spots habit). They also explore how difficult emotions are a shared part of life through a Human Just Like Me exercise (Buddy habit). |
| 3 | Practicing Self-Compassion | Participants dive deeper into both accepting, gentle self-compassion and strong self-compassion (Super Snuggles habit), learn about the 3 For Me practice (i.e., Self-Compassion Break) and learn kind touch. |
| 4 | Opening to Kindness and Compassion | Participants learn about ways to open to kindness and compassion, to send kind wishes, explore why self-compassion can be tricky, and learn a guided visualization called Compassionate Friend (Super Snuggles and Sunny habits). |
| 5 | Meeting Difficult Emotions | Participants discuss strengths and weaknesses, anger, universal needs (the Buddy habit), soft feelings underneath anger, and learn a Squeeze and Soften exercise (Doodles habit). |
| 6 | Embracing Your Life | Participants discuss how to soak in good things, develop self-appreciation, and explore gratitude (Sunny habit). They are led through a Custom Kind Wishes practice and review course content, discussing ways to remember and practice going forward (including all Resilience Habit Animals). |
| *Note*. Five Resilience Habit Animals are used throughout the course to make the content playful and accessible for children. Spots the giraffe helps children notice their emotions, thoughts, body sensations, and five senses. Buddy the dog helps children know they are not alone. Super Snuggles the bunny offers tender kindness and strong support. Doodles the dolphin helps children take kind and helpful actions. Sunny the sun helps children notice and soak in good things (Sunny is grouped with the animals for conceptual consistency). The habits are referred to by their animal names throughout the course (e.g., reminding yourself you are not alone is referred to as “the Buddy habit”). | | |

**Appendix A**. Reflective Functioning Definition and Coding Framework

**REFLECTIVE FUNCTIONING (RF)**

Defined as the ability to recognize and understand emotions in the self and others (mentalizing) and how emotions affect behavior. This includes the ability to recognize other’s needs, including when these needs are not obvious or directly communicated. RF is assessed by having individuals discuss a range of emotional experiences and these narratives are coded according to the individuals’ ability to discuss and reflect on their emotional experiences (Fonagy, Steele, Moran, Steele, & Higgitt, 1991).

**A1**. Reflective Functioning Rating Scale (-1 to 7)

Each interview question is coded on a scale from –1 to 7, reflecting increasing levels of mentalizing capacity.

Note: Mental States include thoughts, feelings, desires, and motivations. Verbs such as “thought, felt, needed, wanted, wished for, hoped for, liked, hated” or feeling words (e.g., annoyed, sad, anxious) can indicate a mental state.

| **Scale Score** | **Description** | **Example Quote** |
| --- | --- | --- |
| **-1 (extreme)** | Passage shows distortion or active antagonizing | He was not actually sad; he just wanted attention. |
| **1**  **(low)** | Passage shows disavowal or a response devoid of content | I don’t know what he was thinking or feeling. |
| **2**  **(low)** | Passage describes a behavior or personality trait | He was working hard. |
| **3**  **(low)** | Passage shows a simple mention of single mental state | She was probably feeling frustrated. |
| **4**  **(moderate)** | Passage mentions multiple mental states. There is an ambiguous link between mental states and behaviors, or between two mental states | She was probably embarrassed. She doesn’t like being wrong. |
| **5**  **(moderate)** | Passage shows single link between two mental states or between a mental state and behavior.  Alternatively, passage acknowledges limits of knowing another’s mental state, acknowledges that others can hide their mental state, or accurately attributes a behavior to an underlying mental state. | I was feeling nervous for her and so I kept trying to help her figure out the puzzles. |
| **6**  **(high)** | Passage shows a single link between a mental state and a behavior or two mental states. Multiple mental states are mentioned. | I think she felt frustrated and embarrassed. The more frustrated she became, the more anxious I became. |
| **7**  **(very high)** | Passage shows multiple links between a mental state and a behavior or two mental states. Multiple examples of mentalizing are demonstrated. | I am not sure exactly what she was feeling. I think she was likely embarrassed, but she was pretending it was not a big deal that she was not getting any right. I could tell she was also annoyed and frustrated because she was clicking the mouse vigorously and not answering me. |

**Appendix B**. Reflective Functioning (RF) Summary Interview Guide

RF scores are calculated as the average rating across relevant questions.

**B1**. Summary Interview Guide for Child

**Self-Focused RF (Average of):**

1. What were you thinking about while you worked on the puzzles?
2. What were you thinking about how you did on the puzzles?
3. How did you feel while you were working on the puzzles?
4. How do you feel that you did on the puzzles?

**Caregiver-Focused RF (Average of):**

1. How do you think your [caregiver] felt as they were watching you work on them?
2. What do you think they were thinking about while you were working on them?
3. How do you think your [caregiver] felt about how you did on the puzzles?

**B2**. Summary Interview Guide for Caregiver

Self-Focused RF (Average of):

1. What were you thinking while [child] worked on the puzzles?
2. What were you thinking about how [child] did on the puzzles?
3. And how did you feel watching [child] work on the puzzles?
4. And how did you feel about how [child] did on the puzzles?

Caregiver-Focused RF (Average of):

1. What do you think [child] was thinking while they were working on those puzzles?
2. What do you think [child] was thinking about how they did on the puzzles?
3. How do you think [child] felt while they were working on the puzzles?
4. How do you think [child] felt about how they did on the puzzles?

**Appendix C**. Video Coding Scheme: Support and Overcontrol Scales

**Behavioral Involvement Parenting Scale (BIPS) Coding System**. Rasmussen, H.F., & Borelli, J.L. Unpublished Coding Manual.

| **Categorization (Support or Overcontrol)** | **Description** | **Coding Score** |
| --- | --- | --- |
| Support | Caregiver praises child success or partial success. | (Yes=1)  (No=0) |
|  | Caregiver makes supportive type statements to child during the video. |  |
|  | Caregiver avoids making general negative or uncomplimentary remarks about the child. |  |
|  | Caregiver smiles/nods in response to child’s task performance. |  |
|  | Caregiver responds to child’s vocalizations with a verbal response. |  |
|  | Caregiver gently pats, caresses, strokes, hugs, touches, or kisses child during or after puzzle completion. |  |
|  | Caregiver praises child’s efforts or behaviors broadly (in general). |  |
|  | Caregiver comforts their child. |  |
| Overcontrol | Caregiver makes critical or negative comments about the child’s task performance. | (Yes=1)  (No=0) |
|  | Caregiver vocalizes during task not in response to child’s vocalization. |  |
|  | Caregiver touches child to provide direction on puzzle completion. |  |
|  | Caregiver touches or points to the computer to provide direction on puzzle completion. |  |
|  | Caregiver yells at the child during the video. |  |
|  | Caregiver uses imperative verbal style more than explanatory when talking about the puzzles. |  |
|  | Caregiver gives verbal instructions during the puzzle. *(Any verbal instruction would score a yes here unless in response to child’s explicit verbal request for help.)* |  |
|  | Caregiver intrudes on child’s attempt at the puzzle *(within first 5 seconds).* |  |
|  | Mom moves closer to the child during the puzzle. |  |
